# Racial differences in COVID-19 vaccine acceptance in Arkansas

**DOI:** 10.1371/journal.pone.0268876

**Published:** 2023-05-18

**Authors:** Benjamin C. Amick III, Jaimi L. Allen, Clare C. Brown, Anthony Goudie, Mick Tilford, Mark Williams

**Affiliations:** 1 Department of Epidemiology, Fay W. Boozman College of Public Health, University of Arkansas for Medical Sciences, Little Rock, Arkansas, United States of America; 2 Winthrop P. Rockefeller Cancer Institute, University of Arkansas for Medical Sciences, Little Rock, Arkansas, United States of America; 3 Department of Health Policy and Management, Fay W. Boozman College of Public Health, University of Arkansas for Medical Sciences, Little Rock, Arkansas, United States of America; 4 Department of Health Education and Health Behavior, Fay W. Boozman College of Public Health, University of Arkansas for Medical Sciences, Little Rock, Arkansas, United States of America; Iran University of Medical Sciences, ISLAMIC REPUBLIC OF IRAN

## Abstract

Vaccines are one of the most successful tools for protecting the public’s health. However, widespread vaccine hesitancy in the Southern United States is preventing effective mitigation of the current COVID-19 pandemic. The purpose of this study was to assess COVID-19 vaccine acceptance among adults living in a largely rural Southern state. This cross-sectional study collected data from 1,164 Arkansas residents between October 3 and October 17, 2020 using random digit dialing. The primary outcome was a multidimensional COVID-19 vaccine acceptance measure with scores between -3 to +3. The full COVID-19 vaccine acceptance scale was measured along with perceived vaccine safety, effectiveness, acceptance, value, and legitimacy subscales. Statistical analyses were conducted using multivariable linear regression. Results indicated Black participants had the lowest overall vaccine acceptance (0.5) compared to White participants (1.2). Hispanic participants had the highest scores (1.4). In adjusted models, Black participants had 0.81 points lower acceptance than White participants, and Hispanic participants had 0.35 points higher acceptance. Hispanic participants had the highest scores for all five vaccine acceptance subscales, relatively equivalent to White participants. Black participants had consistently lower scores, especially perceived vaccine safety (mean -0.2, SD 0.1). In conclusion, the lowest vaccine acceptance rates were among Black participants particularly on perceived vaccine safety. While Black participants had the lowest acceptance scores, Hispanic participants had the highest. This variability shows the value of a multidimensional vaccine acceptance measure to inform COVID-19 vaccination campaign strategies.

## Introduction

More than 99.7 million Americans have contracted COVID-19 and over 1.08 million have died from the disease as of December 2022 [1]. In Arkansas specifically, 979,282 COVID-19 cases and 12,667 deaths have been reported as of December 2022 [2]. Marginalized communities have been disproportionally impacted by the pandemic. Racial/ethnic minority populations experience higher rates of COVID-19-related illness, COVID-19 case rates, hospitalizations, and deaths, and a larger life expectancy loss when compared to white counterparts [3–7]. One study conducted in the earlier stages of the pandemic (April 2020) found that although counties considered majority black only make up approximately 20% of counties in the U.S., they accounted for 52% of COVID-19 diagnoses and 58% of COVID-19 deaths nationally [3]. An observational cohort study conducted in May 2020 in Louisiana was one of the first states to publish COVID-19 statistics according to race [4]. They found that even though black residents only make up 31% of the population, 76.9% of the hospitalized patients with COVID-19 and 70.6% of those who died were black [4].

Recent data released in August 2022 indicates widened and narrowed disparities in COVID-19 infections and deaths at various time points throughout the pandemic. However, total cumulative COVID-19 case and death rates and hospitalizations are higher among racial/ethnic minorities when compared to White people when adjusted to account for differences in age by race and ethnicity [5]. Cumulative COVID-19 age-adjusted infection rates in order from highest to lowest show the following rates per 100,000 population: Native Hawaiian or Other Pacific Islander (NHOPI; 22,116), Hispanic (21,863), American Indian or Alaska Native (AIAN; 19,917), Black (15,639), White (14,858), and Asian (11,555) [4]. It is important to note that all case data are likely significantly underreported since home testing is not reported to public health agencies [5]. Risk for COVID-19 infection, hospitalization, and death is also higher for all racial and ethnic minority groups when compared to White, non-Hispanic (NH) persons with the exclusion of NH Asian persons [6]. Rate ratios for cases, hospitalizations, and deaths when compared to NH White persons are as follows: AIAN (1.6x, 2.5x, 2.1x), Hispanic (1.5x, 1.9x, 1.8x), and Black (1.1x, 2.2x, 1.7x) [6]. Significant differences in life expectancy by race has also been observed as a result of the pandemic. Most high-income countries experienced losses in life expectancy in 2020 and 2021 [7, 8], but recent data shows an uneven pandemic-related impact by race/ethnicity in the United States [9]. Although NH White persons saw a 1.2-year decline in life expectancy in 2020, Hispanic populations saw a 3-year decline and Black persons saw a 2.9-year decline [9].

Even though vaccines are one of the most successful tools for protecting the public’s health [10], vaccine hesitancy has resulted in uncertainty in COVID-19 vaccine uptake [11]. Four COVID-19 vaccines (Pfizer-BioNTech, Moderna, Janssen, and Novavax, Adjuvanted) have received full approval or emergency use authorization from the Food and Drug Administration (FDA) and have been distributed throughout the United States [12]. Just recently, the FDA authorized the bivalent COVID-19 vaccine for children beginning at 6 months of age [12]. While nearly 69% of Americans have completed the primary series of the vaccine, less than 57% of Arkansans have been fully vaccinated [13]. Similar trends are seen with the updated (bivalent) booster dose with 14% of the U.S. population ≥5 years of age compared to 9% in Arkansas [13]. Even with effective COVID-19 vaccines, there is little expectation Americans will accept the vaccine in sufficient numbers to achieve herd immunity [14, 15]. Mistrust and doubts plaguing vaccinations in general [16] prevent the acceptance of a COVID-19 vaccine [17].

Earlier research addressing vaccine acceptance among racial/ethnic minorities showed lower rates of flu vaccination among Black individuals and higher levels of resistance to the flu vaccine [18, 19]. Similar trends were initially seen among racial/ethnic minority populations after the COVID-19 vaccine rollout. By the end of April 2021, Asian and NH White adults had the highest COVID-19 vaccine uptake; however, these disparities narrowed for some groups by November 2021 with similar coverage for Hispanic, NH White, NH Black, and NHOPI populations (81–76%) [20]. It is important to note that current COVID-19 vaccination trends by race/ethnicity are significantly underestimated since race/ethnicity is not available for approximately 25% of those who have received at least one dose of the vaccine [13]. To prevent adding to healthcare disparities already highlighted during the pandemic, we need to better understand how different communities of color feel about the COVID-19 vaccine and how we can personalize vaccine campaign strategies. At the time of the study, Black adults made up just 12% of Arkansans who had received both vaccine doses, even though they accounted for 15% of the state’s COVID-19 deaths [2] and 16% of the state’s population [21]. Updated information indicates 13.1% of vaccinations were administered to Black Arkansans, and 15.6% of the total cases and 13.9% of deaths were among Black Arkansans [2].

In this paper, we examine vaccine acceptance among a random sample of Arkansas adults and explore differences by race/ethnicity. Vaccine acceptance is the willingness to receive a vaccine. We explore the utility of a multidimensional scale and whether using a multidimensional scale provides insights into racial/ethnic disparities in vaccine acceptance. Risk factors for racial/ethnic differences in COVID-19 vaccine acceptance include other social determinants of health (e.g., socioeconomic status, community vulnerability, education, occupation, structural discrimination), psychosocial factors (e.g., discrimination, health literacy), health care access issues, and biological factors (e.g., age) [6]. Thus, study results can inform vaccine campaign strategies, enhancing equitable access to and receipt of COVID-19 vaccinations and boosters. Knowledge surrounding racial and ethnic disparities is critical to reducing disparities in vaccination uptake.

## Materials and methods

### Study population

This cross-sectional study collected data from 1,536 Arkansas residents between October 3 and October 17, 2020. There was no racial or gender bias in participant selection. Participants were contacted using random digit dialing of mixed ground line and targeted cell phone numbers in Arkansas. Land lines were a random sample of all known land lines in Arkansas. Cell phone numbers were a random sample of active numbers used in Arkansas. Usage in the state was determined by the volume of calls and locations where a particular cell phone was used most. This target approach eliminated cell numbers not currently in use or used outside the state at the time of the poll. Additionally, counties with higher Hispanic and Black populations were over-sampled. The mixed sample of ground and cell phones was purchased from a national company (Dynata Inc.) having access to these data and experience doing telephone polling in Arkansas. Samples of phone numbers were received from the company every two weeks.

### Participant recruitment and ethical consideration

Trained research assistants (RAs) called telephone numbers from a randomized non-duplicative list. Respondents had to be an adult (18 years of age or older) and an Arkansas resident to be eligible for inclusion in the study. If the person answering the call met study eligibility criteria, the RA explained they were calling from a health science center and asked if the respondent was interested in answering questions about the COVID-19 pandemic. The RA then explained what participation in the study entailed, including (1) the estimated time (15 min); (2) potential risks and benefits; (3) the voluntary nature of participation; and (4) confidentiality of responses. Respondents indicated their consent by agreeing to participate in the survey. The average length of interview was 11 minutes. The questionnaire included 40 questions on sociodemographic characteristics and COVID-19 vaccine acceptance. Respondents could refuse to answer any question on the survey, or state they did not know, and still continue the survey. If the person refused to participate or the phone was not answered, the RA proceeded to the next number. In the event the person reached was only Spanish speaking, an RA fluent in Spanish spoke with the respondent. Study procedures were approved by an institutional review board for the protection of human subjects (# 260974).

### Measures

Vaccine acceptance was measured using the 10-item short version of a multi-component measure of vaccine acceptance developed by Sarathchandra et al. [22]. This measure was developed for measuring the full conceptual dimensionality of vaccine acceptance in the general public. Study participants were asked to focus on a COVID-19 vaccine when answering the questions. The scale is composed of five subscales (two items each) measuring distinct dimensions of vaccine acceptance (see S1 Dataset). The five dimensions are: perceived safety of a vaccine (safety); perceived vaccine effectiveness and necessity (effective); acceptance of vaccine selection and scheduling of doses (accept); perceived value of and affect toward the vaccine (value); and the perceived legitimacy of authorities to require people be vaccinated (legitimate). Responses were recorded on a seven-point Likert-type scale ranging from one (strongly disagree) to seven (strongly agree). The mid-point of the scale was neither disagree nor agree. To arrive at a vaccine acceptance score, negatively worded questions were reverse scored, and all items were recoded to create a scale ranging from negative three (disagree) to positive three (agree) with zero as the midpoint. The overall scale’s Cronbach’s alpha for the 10 items measuring vaccine acceptance was high at 0.83, and 0.85 to 0.91 for each of the five subscales [22]. In this study, we explore overall vaccine acceptance and each subscale by sociodemographic characteristics and COVID-19 attitudes, mitigation behaviors, and actions taken by the state. Overall vaccine acceptance was then explored and stratified by the two prominent racial categories in our sample (White and Black adults).

Study respondents were asked to provide their gender (male/female), age, race/ethnicity (schema described below), household income in 2019, and Arkansas county of residence. Participants were asked several questions related to attitudes and beliefs about COVID-19, prevention behaviors, and actions taken by the state government to stop the spread of the virus in the state. Among these were: perceived chances of getting COVID-19 (no chance = 0 and any chance = 1), regularly wear a face mask (no/yes), perception that face masks help to control the spread of COVID-19 (no/yes), agreement with a needed state mask order (no/yes), and attending a religious or church service in person in the past 30 days (no/yes). Respondents were also asked to rank on a scale of zero to five, with zero being least and five being strongest, their feelings about the importance of being tested, feeling safe while shopping or eating in public, and optimism of the pandemic ending soon. Finally, respondents were asked if they had health insurance (yes/no).

### Statistical analysis

Data were collected using computer assisted telephone interviews with data stored in a Research Electronic Data Capture (RedCap) database. Stata version SE 16.1 (StataCorp LLC, College Station, TX) was used to manage and analyze data. No duplicate records were discovered in the data management process. The degree of missing data and whether data were missing at random was examined for all variables. Of the 1,536 observations, 140 observations with three or more total missing values or any one missing value on the ten-item vaccine acceptance scale were dropped. Regression diagnostics for outliers indicated 186 outliers with an influence on regression results, so a decision was made to drop observations with a residual absolute value exceeding 2.0, resulting in a final sample size of 1,164. Tests for heteroscedasticity, multicollinearity, and specification error did not reveal significant problems. To explore racial differences, we stratified the regression results for Black and White participants. Hispanic and ‘Other’ racial categories were too small to allow presentation of stratified results.

To ensure the sample was representative, responses were weighted for age, sex, and White/non-White race/ethnicity. Weights were generated using a raking ratio estimation. Population percentages were generated using census data [23]. The population weights were the ratio of percentages observed from the expected in each of the three-way cross-tabulated categories. In preliminary analyses, distributional characteristics of variables were examined. Due to small numbers, Pacific Islander/Marshallese (0.3%), Asian (0.6%), Native American (0.9%), and other (2%) were collapsed into a single “other” category. Final racial/ethnic categories included in analyses were: White/Caucasian, Black/African American, Hispanic/Latino, and other. Age groups were 18 to 29, 30 to 39, 40 to 49, 50 to 59, 60 to 69, and 70 and above. Overall COVID-19 vaccine acceptance scores were calculated including five subscales (safety, effectiveness, acceptance, value, and legitimacy). Calculations ranged from negative three (-3) to three (+3) with zero as the midpoint.

Multivariable linear regression analyses were used to examine the influence of sociodemographic characteristics (age, sex, race/ethnicity, income, region) and pandemic mitigation attitudes and behaviors (chances of getting COVID-19, regular mask wearing, perception that face masks help, belief that a state face mask order is needed, importance of being tested, feel safe going shopping or eating out, optimism pandemic will end soon, attends social gatherings, attends in-person religious services, and health insurance status) on vaccine acceptance. In preliminary analyses, all variables showing statistical (p ≤ 0.20) or conceptual importance were retained in the final multivariable models. Consequently, region of the state, optimism, and feeling safe were removed from the final regression models. A statistical significance level of p ≤ 0.05 was used in final variable selection. All statistically significant results were evaluated to determine if differences were practically significant. Retained variables include: age, gender, race, income, perceived chances of getting COVID-19, regular mask wearing, belief that masks help stop COVID-19 spread, belief that a state face mask order is needed, importance of being tested, attends social gatherings, attends in-person religious services, and has health insurance.

## Results

We present the weighted descriptive statistics for the sociodemographic characteristics in Table 1. The average age of respondents was 45.5 years, and the majority were women (61%). Racial categories included White (75%), Black (16%), Hispanic (4%), and Other (5%). Most reported an unknown household income (32%), followed ≤$30,000 (20%), $31–50,000 (16%), $51–75,000 (12%), and $76–100,000 (10%). Participants were spread throughout the state to include Central (32%), Northwest (28%), Northeast1(19%), Southeast (12%), and Southwest (9%) regions.

**Table 1:** Mean vaccine acceptance (overall and subscales) scores by sociodemographic characteristics.

	Total	Overall Vaccine Acceptance Scale	Vaccine Acceptance Subscales				
			Safety	Effective	Accept	Value	Legitimate
	*n* (%)	Mean (SD)					
Sample		1.1 (1.1)	.71 (1.6)	1.1 (1.4)	1.1 (1.5)	1.4 (1.4)	1.0 (1.6)
Age							
18–29	172 (15%)	1.0 (1.2)	.8 (1.7)	1.0 (1.5)	1.1 (1.5)	1.2 (1.6)	.9 (1.6)
30–39	203 (17%)	1.1 (1.2)	.8 (1.7)	1.0 (1.5)	1.2 (1.5)	1.5 (1.4)	1.0 (1.6)
40–49	189 (16%)	1.2 (1.1)	.9 (1.6)	1.3 (1.3)	1.3 (1.5)	1.6 (1.4)	1.0 (1.6)
50–59	139 (12%)	1.0 (1.1)	.6 (1.7)	1.1 (1.5)	1.0 (1.6)	1.3 (1.4)	1.0 (1.5)
60–69	223 (19%)	1.1 (1.1)	.6 (1.6)	1.1 (1.3)	1.1 (1.5)	1.4 (1.3)	1.1 (1.7)
70+	238 (20%)	1.1 (1.0)	.7 (1.4)	1.1 (1.3)	1.1 (1.4)	1.3 (1.4)	1.1 (1.5)
Sex							
Male	459 (39%)	1.0 (1.1)	.7 (1.6)	1.0 (1.4)	1.1 (1.5)	1.4 (1.4)	.9 (1.6)
Female	705 (61%)	1.1 (1.1)	.7 (1.6)	1.2 (1.4)	1.2 (1.5)	1.4 (1.4)	1.1 (1.6)
Race							
White	876 (75%)	1.2 (1.1)	.9 (1.6)	1.2 (1.4)	1.2 (1.5)	1.5 (1.4)	1.1 (1.6)
Black	187 (16%)	.5 (1.0)	-.2 (1.5)	.6 (1.4)	.6 (1.5)	.7 (1.4)	.7 (1.6)
Hispanic	46 (4%)	1.4 (1.0)	1.1 (1.6)	1.5 (1.2)	1.6 (1.3)	1.5 (1.4)	1.4 (1.4)
Other	55 (5%)	1.0 (1.1)	.6 (1.5)	1.1 (1.4)	1.2 (1.6)	1.3 (1.3)	.8 (1.7)
Household income							
≤30,000	235 (20%)	.9 (1.0)	.5 (1.6)	1.0 (1.3)	1.0 (1.4)	1.1 (1.4)	.9 (1.6)
31–50,000	182 (16%)	1.0 (1.1)	.6 (1.6)	.9 (1.4)	1.0 (1.6)	1.2 (1.5)	1.0 (1.7)
51–75,000	142 (12%)	1.1 (1.1)	.8 (1.7)	1.1 (1.4)	1.2 (1.6)	1.6 (1.4)	.9 (1.6)
76–100,000	114 (10%)	1.5 (1.1)	1.4 (1.6	1.6 (1.5)	1.5 (1.5)	1.9 (1.4)	1.3 (1.6)
≥100,000	122 (10%)	1.7 (1.0)	1.3 (1.5)	1.6 (1.3)	1.8 (1.3)	2.2 (1.1)	1.5 (1.6)
Don’t know	369 (32%)	.9 (1.0)	.5 (1.5)	.9 (1.3)	.9 (1.4)	1.2 (1.3)	.9 (1.5)
Region							
Central	369 (32%)	1.1 (1.1)	.8 (1.6)	1.2 (1.4)	1.1 (1.5)	1.4 (1.4)	1.0 (1.6)
Northeast	221 (19%)	1.0 (1.1)	.6 (1.7)	.9 (1.4)	1.1 (1.5)	1.3 (1.5)	1.0 (1.5)
Northwest	323 (28%)	1.2 (1.0)	.9 (1.5)	1.2 (1.4)	1.2 (1.5)	1.6 (1.3)	1.2 (1.5)
Southeast	136 (12%)	1.0 (1.1)	.5 (1.6)	.9 (1.3)	1.1 (1.4)	1.3 (1.4)	1.0 (1.7)
Southwest	109 (9%)	.9 (1.0)	.6 (1.7)	1.0 (1.4)	1.2 (1.6)	1.2 (1.5)	.7 (1.7)
	N = 1,164 *could be lower due to missing values. Range of scores: -3 to 3.						

Mean scores of vaccine acceptance by sociodemographic characteristics are also reported in Table 1. Female respondents were slightly more accepting of the vaccine and for perceived effectiveness, selection, and legitimacy of authorities to require vaccinations subscales. However, results indicate noticeable differences by race and income. White and Hispanic respondents were more accepting compared to Black and ‘other’ respondents for each subscale. Black participants had the only mean score less than zero, which was on the vaccine safety subscale. Vaccine acceptance scores tended to increase as income increased, indicating higher income respondents were more accepting of a COVID-19 vaccine.

Table 2 shows mean scores of vaccine acceptance by COVID-19 attitudes, mitigation behaviors, and actions taken by the state. Nearly 1/4 (22%) of respondents believed they had no chance of contracting COVID-19, but most respondents (93%) reported regularly wearing a face mask. Only 24% of respondents felt like face masks help stop the spread of COVID-19. Most (69%) respondents felt a state face mask order was needed, and 81% believed it was important to be tested for the virus. Most respondents felt safe shopping or eating out (72%), but approximately ¾ of respondents did not attend gatherings of 10 people or more (75%) nor did they attend in-person religious services (74%). A little more than ½ (56%) of the respondents were optimistic the pandemic would end soon. The majority of participants (93%) had health insurance.

**Table 2:** Mean vaccine acceptance (overall and subscales) scores by COVID-19 attitudes, mitigation behaviors, and actions taken by the state.

	Total	Overall Vaccine Acceptance Scale	Vaccine Acceptance Subscales				
			Safety	Effective	Accept	Value	Legitimate
	*n* (%)	Mean (SD)					
Sample		1.1 (1.1)	.71 (1.6)	1.1 (1.4)	1.1 (1.5)	1.4 (1.4)	1.0 (1.6)
Chances of getting COVID-19							
Any chance	909 (78%)	1.2 (1.1)	.8 (1.6)	1.2 (1.3)	1.2 (1.5)	1.5 (1.4)	1.1 (1.5)
No chance	255 (22%)	.7 (1.1)	.4 (1.7)	.7 (1.5)	.9 (1.6)	1.0 (1.5)	.6 (1.7)
Regularly wears a mask in public							
Yes	1,087 (93%)	1.1 (1.1)	.8 (1.6)	1.1 (1.4)	1.2 (1.5)	1.4 (1.4)	1.1 (1.5)
No	77 (7%)	.1 (1.1)	-.4 (1.8)	.4 (1.3)	.4 (1.6)	.6 (1.5)	-.4 (1.7)
Face masks help stop COVID-19 spread							
Yes	888 (76%)	1.2 (1.0)	.9 (1.5)	1.2 (1.3)	1.2 (1.4)	1.5 (1.4)	1.2 (1.5)
No	276 (24%)	.6 (1.2)	.1 (1.7)	.6 (1.4)	.8 (1.7)	1.1 (1.5)	.3 (1.7)
State face mask order is needed							
Yes	800 (69%)	1.2 (1.1	.8 (1.5)	1.2 (1.3)	1.2 (1.4)	1.4 (1.4)	1.2 (1.5)
No	364 (31%)	.9 (1.1)	.5 (1.7)	.9 (1.4)	1.0 (1.6)	1.3 (1.5)	.7 (1.7)
Importance of being tested							
Important	947 (81%)	1.1 (1.1)	.8 (1.5)	1.1 (1.4)	1.2 (1.5)	1.4 (1.4)	1.1 (1.5)
Not important	217 (19%)	.8 (1.2)	.3 (1.8)	.9 (1.4)	.9 (1.6)	1.2 (1.4)	.5 (1.7)
Feel safe shopping / eating out							
Safe	828 (72%)	1.0 (1.1)	.7 (1.6)	1.0 (1.4)	1.1 (1.5)	1.4 (1.4)	.9 (1.6)
Unsafe	328 (28%)	1.2 (1.1)	.7 (1.6)	1.2 (1.3)	1.3 (1.4)	1.4 (1.4)	1.2 (1.5)
Optimism pandemic will end soon							
Optimistic	511 (44%)	1.0 (1.1)	.6 (1.7)	1.0 (1.4)	1.1 (1.5)	1.2 (1.4)	.8 (1.7)
Not optimistic	651 (56%)	1.2 (1.1)	.8 (1.6)	1.2 (1.4)	1.2 (1.5)	1.5 (1.4)	1.2 (1.5)
Attended large social gatherings (10+ people) in past 2 weeks							
Yes	290 (25%)	1.1 (1.2)	.7 (1.7)	1.1 (1.4)	1.2 (1.5)	1.5 (1.4)	.9 (1.7)
No	874 (75%)	1.1 (1.1)	.7 (1.6)	1.1 (1.4)	1.1 (1.5)	1.4 (1.4)	1.1 (1.6)
Attended in-person religious services in past 2 weeks							
Yes	304 (26%)	.9 (1.2)	.5 (1.7)	1.0 (1.4)	1.1 (1.6)	1.3 (1.5)	.8 (1.7)
No	860 (74%)	1.1 (1.1)	.8 (1.6)	1.1 (1.4)	1.2 (1.5)	1.4 (1.4)	1.1 (1.6)
Health insurance							
Yes	1,088 (93%)	1.1 (1.1)	.8 (1.6)	1.1 (1.4)	1.2 (1.5)	1.4 (1.4)	1.1 (1.6)
No	76 (7%)	.6 (1.3)	.1 (1.7)	.6 (1.5)	.8 (1.8)	1.0 (1.6)	.4 (1.8)
	N = 1,164 *could be lower due to missing values. Range of scores: -3 to 3.						

When exploring vaccine acceptance by COVID-19 attitudes and behaviors, noticeable differences can be seen in perceived chances of getting COVID-19, regular face mask wearing, believing masks help stop the spread of COVID-19, importance of being tested, feeling safe when shopping or eating out, and having health insurance. Participants who indicated they did not regularly wear a face mask had the only mean scores below zero (-0.4), indicating dismissal of vaccine safety and legitimacy. Those who indicated a perceived risk of acquiring COVID-19 were more accepting of the vaccine overall and each subscale when compared to those who indicated they had no chance of getting COVID-19 (overall: 1.2 vs. 0.7; safety: 0.8 vs. 0.4; effectiveness: 1.2 vs. 0.7; acceptance: 1.2 vs. 0.9; value: 1.5 vs. 1.0; legitimacy: 1.1 vs. 0.6). Additionally, the following categories were more accepting of the vaccine overall and for each subscale: regular face mask wearers and those who believed face masks help stop COVID-19 spread, a state face mask order is needed, being tested for COVID-19 is important, not optimistic pandemic will end soon, does not attend religious services, and has health insurance.

Results from adjusted multivariable linear regression models are shown in Table 3. When looking at overall vaccine acceptance in the first column, results indicate Black participants (-0.81, p ≤ .001) were significantly less accepting of a COVID-19 vaccine, while Hispanic participants (0.35, p = .02) were more accepting compared to non-Hispanic Whites. Those with a household income of more than $51,000 ($51–75,000: 0.24, p = .02; $76–100,000: 0.71, p ≤ .001; ≥$101,000: 0.85, p ≤ .001) were more accepting than those with a household income of $30,000 or less. Several COVID-19 attitudes and behaviors had a significant positive association with vaccine acceptance: perceived chances of getting COVID-19 (0.15, p = .04), regularly wearing a mask (0.79, p ≤ .001), belief that masks help stop the spread of COVID-19 (0.41, p ≤ .001), believed being tested is important (0.18, p = .02), and had health insurance (0.42, p = .04). Attending church or religious services in person (-0.16, p = .02) was the only factor showing a negative association with vaccine acceptance.

**Table 3:** Linear regression of vaccine acceptance on sociodemographic characteristics and COVID-19 attitudes and behaviors for the overall population and for black and white adults.

Variable	Overall (n = 1,164)	White (n = 876)	Black (n = 187)
Intercept	-1.03 (.22)	-1.23 (.25)	-.34 (1.16)
	Odds Ratio (Standard Error; 95% Confidence Interval)		
Age (reference [ref]: 18–29)			
30–39	.01 (.10; -0.18–0.21)	-.06 (.12; -0.3–0.17)	.22 (.24; -0.25–0.69)
40–49	.11 (.10; -0.09–0.31)	.08 (.12; -0.16–0.33)	.12 (.24; -0.36–0.6)
50–59	-.03 (.11; -0.25–0.19)	-.05 (.14; -0.32–0.22)	.25 (.25; -0.25–0.75)
60–69	-.04 (.10; -0.24–0.15)	-.10 (.12; -0.33–0.13)	.27 (.24; -0.21–0.74)
70+	.00 (.10; -0.2–0.2)	-.04 (.11; -0.26–0.19)	.35 (.25; -0.14–0.83)
Sex (ref: male)			
Female	.09 (.06; -0.02–0.21)	.03 (.07; -0.1–0.16)	.48** (.14; 0.19–0.76)
Race (ref: White)			
Black	-.81** (.08; -0.96–0.65)		
Hispanic	.35* (.15; 0.05–0.64)		
Other	-.16 (.13; -0.42–0.1)		
Household income (ref: ≤30,000)			
31–50,000	.18 (.10; -0.01–0.36)	.16 (.11; -0.06–0.38)	.33 (.20; -0.06–0.72)
51–75,000	.24* (.10; 0.04–0.44)	.23* (.12; 0.0–0.47)	.22 (.25; -0.28–0.71)
76–100,000	.71** (.11; 0.49–0.94)	.76** (.13; 0.51–1.0)	.24 (.32; -0.39–0.88)
100,000 or more	.85** (.11; 0.64–1.07)	.86** (.13; 0.6–1.1)	.87** (.27; 0.34–1.39)
Don’t know	.01 (.08; 0.15–0.17)	.01 (.09; -0.18–0.19)	.15 (.18; -0.19–0.5)
Any chance of getting COVID-19	.15* (.07; 0.01–0.29)	.10 (.08; -0.06–0.27)	.50** (.16; 0.19–0.8)
Regular mask wearer	.79** (.13; 0.55–1.04)	.82** (.13; 0.56–1.09)	-.73 (.97; -2.65–1.19)
Masks help stop COVID-19 spread	.41** (.08; 0.25–0.57)	.37** (.09; 0.19–0.56)	.47* (.22; 0.04–0.91)
State face mask order is needed	.10 (.07; -0.05–0.24)	.10 (.08; -0.06–0.26)	-.13 (.25; -0.63–0.37)
Importance of being tested	.18* (.08; 0.03–0.34)	.25** (.09; 0.07–0.42)	-.23 (.30; -0.82–0.35)
Attended social gatherings	.10 (.07; -0.04–0.24)	.18* (.08; 0.03–0.34)	-.22 (.19; -0.6–0.16)
Attended religious services in person	-.16* (.07; -0.29–0.03)	-.21** (.08; -0.37–0.06)	.09 (.17; -0.25–0.42)
Has health insurance	.42** (.12; 0.19–0.65)	.61** (.15; 0.31–0.91)	.63* (.30; 0.04–1.22)
	Adj R^2^ = .25	Adj R^2^ = .22	Adj R^2^ = .18

Goodness of fit test: Overall: *F*(22, 1141) = 18.76, *p ˂ 0*.*001*; White: *F*(19, 856) = 14.21, *p ˂ 0*.*001*; Black: *F*(19, 167) = 3.19, *p ˂ 0*.*001*

*/** Indicates significance at the .05/.01 level.

Stratified models shown in the second and third columns of Table 3 indicate differential vaccine acceptance between Black and White respondents. Being a Black woman had a significant positive association with vaccine acceptance (0.48, p ≤ .001). Having a household income above $50,000 had a positive relationship for White participants ($51–75,000: 0.23, p = .05; $76–100,000: 0.76, p ≤ .001; ≥$101,000: 0.86, p ≤ .001), and more than $100,000 for Black participants (0.87, p ≤ .001). Significant racial differences were noted in COVID-19 attitudes and behaviors as well. Regularly wearing a face mask (0.82, p ≤ .001), belief that masks help stop the spread of COVID-19 (0.37, p ≤ .001), belief that it was important to get tested for COVID-19 (0.25, p ≤ .001), and having health insurance (0.61, p = ≤ .001) were found to be significantly related to greater vaccine acceptance for White participants. Attending church or a religious service (-0.21, p = .01) had a negative relationship on vaccine acceptance for White participants only. Higher perceived chances of getting COVID-19 (0.5, p ≤ .01), belief that masks help stop the spread of COVID-19 (0.47, p = .03), and having health insurance (0.63, p = .04) were related to vaccine acceptance among Black participants.

## Discussion

The study investigated COVID-19 vaccine acceptance using a multidimensional scale in the period just proceeding the emergency approval of the vaccine. Evidence on the distribution of COVID-19 vaccine acceptance in populations is emerging yielding important results. At the time of our study, prior to the FDA emergency use authorization of the COVID-19 vaccines, no other known studies had investigated COVID-19 vaccine hesitancy using a multidimensional measure. An earlier study conducted in May 2020 on vaccine acceptance included just over 600 American adults, and more than two thirds of participants reported they would accept a COVID-19 vaccination if recommended for them [24]. Vaccine acceptance was measured by a single item. Results showed significant differences by gender, age, race/ethnicity, education, perception of COVID-19 risk, and region of the United States [14, 24]. The study, however, was limited due to sampling, which required a specific smart phone app to participate. Other studies of vaccine acceptance across the globe have generally found those who perceived themselves at higher risk and had previously been vaccinated for the influenza are more likely to be vaccinated for COVID-19 [17, 24–29]. However, these studies, too, used single measures of vaccine acceptance. At the time of our study, only two known studies in the U.S. explicitly examined racial and ethnic differences in vaccine hesitancy in minority groups compared to non-Hispanic White adults [30, 31]. Longitudinal evidence showed the time during the pandemic when a respondent was asked about intent to get vaccinated (early/late) was associated with degree of uptake [32, 33].

The study was conducted in Arkansas, a small southern rural state. This is relevant because vaccination rates are known to be lower in the rural South, where preventive medical services are generally less available [34–36]. Another cross-sectional study conducted in the southern U.S. recruited participants from various community events in Georgian and South Carolinian cities from December 2020 to April 2021 [37]. Similar to our study, Moore et al. [37] found elevated levels of hesitancy or resistance towards COVID-19 vaccinations among Black adults. Dissimilar to our study, age was the strongest determinant of vaccine hesitancy followed by those experiencing housing insecurity [37].

These results highlight risk factors associated with racial differences in COVID-19 vaccine acceptance as noted by another study investigating the association of discrimination and vaccine hesitancy among Black adults in Arkansas [30]. Their research uncovered heterogeneous attitudes about COVID-19 vaccinations among Black adults. Although a slight majority of Black respondents expressed some vaccine hesitancy, nearly half indicated no hesitancy [30]. Experiences of racial discrimination with police or in the courts was associated with vaccine hesitancy [30]. In fact, these experiences were associated with more than double the odds of hesitancy towards a COVID-19 vaccine [30]. Additional risk factors can include income, education, understanding, and trust in science and healthcare professionals. We found household income to be associated with vaccine acceptance, as those with higher income were more accepting of the vaccine. It is quite likely education also plays a significant role in determining COVID-19 vaccine acceptance. This may be due, as some have suggested, to a basic understanding of and trust in science, rather than acceptance of a COVID-19 vaccine as such [38, 39].

Vaccine hesitancy is not unique to COVID-19. Thus, considering culture is key to understanding vaccine acceptance and subsequent behaviors [40]. Currently, the role of culture on behavioral outcomes, particularly vaccine uptake, is under researched. However, as vaccine hesitancy perpetuates disease progression and deaths, understanding cultural influences are critical to addressing inequitable outcomes [41]. Although the current study is a local (state-specific) study, results may provide insights to cultural influences across regions or states with diverse populations.

In the current study, Black Arkansans had the lowest levels of vaccine acceptance. This is true for the overall vaccine acceptance scale and for each subscale. Lower COVID-19 vaccine acceptance among Black participants may be due to mistrust in the medical establishment and continuing lack of equality in medical services throughout the South [14]. Hispanic and White participants had higher and similar levels of acceptance. These findings strongly suggest a single vaccine acceptance campaign strategy is unlikely to convince large numbers of Black adults to be vaccinated. DeRoo et al. [14] advocate for mobilizing minority community leaders to help motivate minority communities to be vaccinated. In the South, this certainly includes recruiting faith leaders to help motivate their congregations. Distributing the vaccine in churches after services may greatly reduce the costs of visiting a clinic for those dependent on hourly jobs. A key finding in our work is the higher positive acceptance of vaccines among Black women. If one goal is to influence social norms in a community, then relying on Black women, who appear to have more positive attitudes toward the vaccine than Black men, as community opinion leaders may be important in the Black community.

One reason this study was conducted was to examine the value of a more robust conceptualization of vaccine acceptance. The idea of a multidimensional scale is useful if it contributes meaningful variability. The sample is too small to conduct the robust comparisons across subscales, but the measure provided meaningful variability. Only Black participants had a negative valence on the safety subscale. No other subscale produced a negative valence. Hispanic participants have higher levels of vaccine acceptance, higher than White participants on some subscales. We believe this approach to measuring vaccine acceptance or hesitancy is an improvement over a single item measure. As vaccine hesitancy becomes a more significant public health problem, more psychometric research on this key system performance measure should be conducted.

Study strengths include the usage of a multidimensional vaccine acceptance scale and sample size. This study also has some limitations which should be noted. We analyzed cross-sectional data and cannot establish causal relationships. Also, the sample includes the population of only one state. Therefore, findings are generalizable only within Arkansas. Even so, given similarities with the populations of other Southern states, our findings are likely broadly indicative of COVID-19 vaccine acceptance in the South. Additionally, the survey was conducted just before the FDA approved the two mRNA vaccines currently available in the U.S. While the media were reporting the imminent emergency approval of the vaccines while the survey was ongoing, we were unable to assess the acceptance after formal approval of the vaccines.

## Conclusion

Using multidimensional measures, this study shows differences in vaccine acceptance by sociodemographic factors and COVID-19 attitudes and beliefs. A deeper dive into racial differences showed significant differences between Black and White respondents indicating diverse attitudes about a COVID-19 vaccine. Based on our findings, public health efforts should be directed towards individuals who are less likely to accept vaccination. Study results should be considered in policy making for equitable health outcomes and future research. Cultural differences in vaccine hesitancy should be considered in future vaccine rollout efforts, especially in the early stages of a new pandemic. Vaccine acceptance is multidimensional; therefore, researchers should aim to use multidimensional measures when investigating acceptance of emerging vaccines. By using single item measures, conclusions may be drawn, but explanations based on valid measurement may be unattainable. Single measures might also aggregate different populations into a single category, thereby mischaracterizing important differences among groups that can otherwise help personalize public health messaging and campaign strategies. Future studies should retain multidimensional measurements to explore differences among sociodemographic groups in various geographical locations and associated risk factors. Consideration should also be placed on using full measurements, although shortened measurement tools can be more convenient and yield important findings.

## Supporting information

S1 Dataset (XLS)
